# Real-Time Subject-Independent Pattern Classification of Overt and Covert Movements from fNIRS Signals

**DOI:** 10.1371/journal.pone.0159959

**Published:** 2016-07-28

**Authors:** Neethu Robinson, Ali Danish Zaidi, Mohit Rana, Vinod A. Prasad, Cuntai Guan, Niels Birbaumer, Ranganatha Sitaram

**Affiliations:** 1 School of Computer Science and Engineering, Nanyang Technological University, Singapore, Singapore; 2 Max Planck Institute for Biological Cybernetics, Tuebingen, Germany; 3 Institute of Medical Psychology and Behavioral Neurobiology, University of Tuebingen, Germany; 4 Department of Neural and Biomedical Technology, Institute for Infocomm Research, A*STAR, Singapore, Singapore; 5 Wyss Center for Bio and Neuroengineering, Geneva, Switzerland; 6 Department of Psychiatry and Division of Neuroscience, Schools of Engineering, Biology & Medicine, Pontificia Universidad Católica de Chile, Santiago, Chile; University of Sheffield, UNITED KINGDOM

## Abstract

Recently, studies have reported the use of Near Infrared Spectroscopy (NIRS) for developing Brain–Computer Interface (BCI) by applying online pattern classification of brain states from subject-specific fNIRS signals. The purpose of the present study was to develop and test a real-time method for subject-specific and subject-independent classification of multi-channel fNIRS signals using support-vector machines (SVM), so as to determine its feasibility as an online neurofeedback system. Towards this goal, we used left versus right hand movement execution and movement imagery as study paradigms in a series of experiments. In the first two experiments, activations in the motor cortex during movement execution and movement imagery were used to develop subject-dependent models that obtained high classification accuracies thereby indicating the robustness of our classification method. In the third experiment, a generalized classifier-model was developed from the first two experimental data, which was then applied for subject-independent neurofeedback training. Application of this method in new participants showed mean classification accuracy of 63% for movement imagery tasks and 80% for movement execution tasks. These results, and their corresponding offline analysis reported in this study demonstrate that SVM based real-time subject-independent classification of fNIRS signals is feasible. This method has important applications in the field of hemodynamic BCIs, and neuro-rehabilitation where patients can be trained to learn spatio-temporal patterns of healthy brain activity.

## Introduction

In contrast to the classical method of presenting stimuli and studying evoked brain responses, BCI and neurofeedback work by altering the neural activity first, and then observing the effect of this altered activity on the subjects’ behavior [1,2]. This allows the dissection of the functional anatomy of the brain. Furthermore, the ability to learn to volitionally regulate activity from a circumscribed brain area has potential for applications towards rehabilitation. Since most of the evoked responses in the brain are in the form of spatio-temporal patterns of activity (electrical or hemodynamic), a system capable of successfully classifying these patterns is an indispensable tool for rehabilitation [2]. Such a system has been successfully developed for fMRI based BCI using real-time Support Vector Machine (SVM) based classification algorithms [3], and an earlier study has also demonstrated the feasibility of implementing machine learning algorithms in classifying single trial activations using multi-channel fNIRS [4]. Furthermore, feasibility and potential effectiveness of an fNIRS based real-time neurofeedback system on performance of kinesthetic motor imagery has also been reported recently [5]. In this study participants performed motor imagery of finger movements with feedback from relevant cortical signals and irrelevant sham signals. The study showed that true neurofeedback induced significantly greater activation of the contra lateral pre-motor cortex and greater self-assessment scores for kinesthetic motor imagery compared with sham feedback. These results illustrate the efficacy of using both fNIRS signals for neurofeedback and machine-learning algorithms for implementing single-trial classifications from such signals. In the present study, we aimed at combining these two approaches so as to develop a real-time SVM based neurofeedback system based on multi-channel fNIRS signals.

Our first objective was the development of a real-time SVM based pattern classification and neurofeedback system. For our study, we used fNIRS signals from the motor cortex while subjects were performing overt and covert hand movements (movement execution (ME) and movement imagery (MI), respectively). We trained the classifier on patterns evoked during ME and tested them on those evoked during MI, and vice versa, establishing the robustness of the classifier on both modalities. Finally, we determined if a pattern-classifier modeled on a group of subjects could be used to classify activation patterns in a new subject. We trained the generalized, subject-independent classifier on movement execution data of four participants and tested it on both movement imagery and execution in new, untrained participants. The main purpose was to establish that the activation patterns from different subjects can be successfully utilized to generate a group classifier that can then identify similar patterns in new subjects. The most significant application of this technique is toward neurofeedback based neuro-rehabilitation, where such a ‘generalized’ classifier can be trained on spatio-temporal activation patterns of healthy subjects, and then used to help train patients to ‘modulate’ their activity to represent healthy activation patterns.

## Methods

### Experiment Protocol

**Fig 1:**
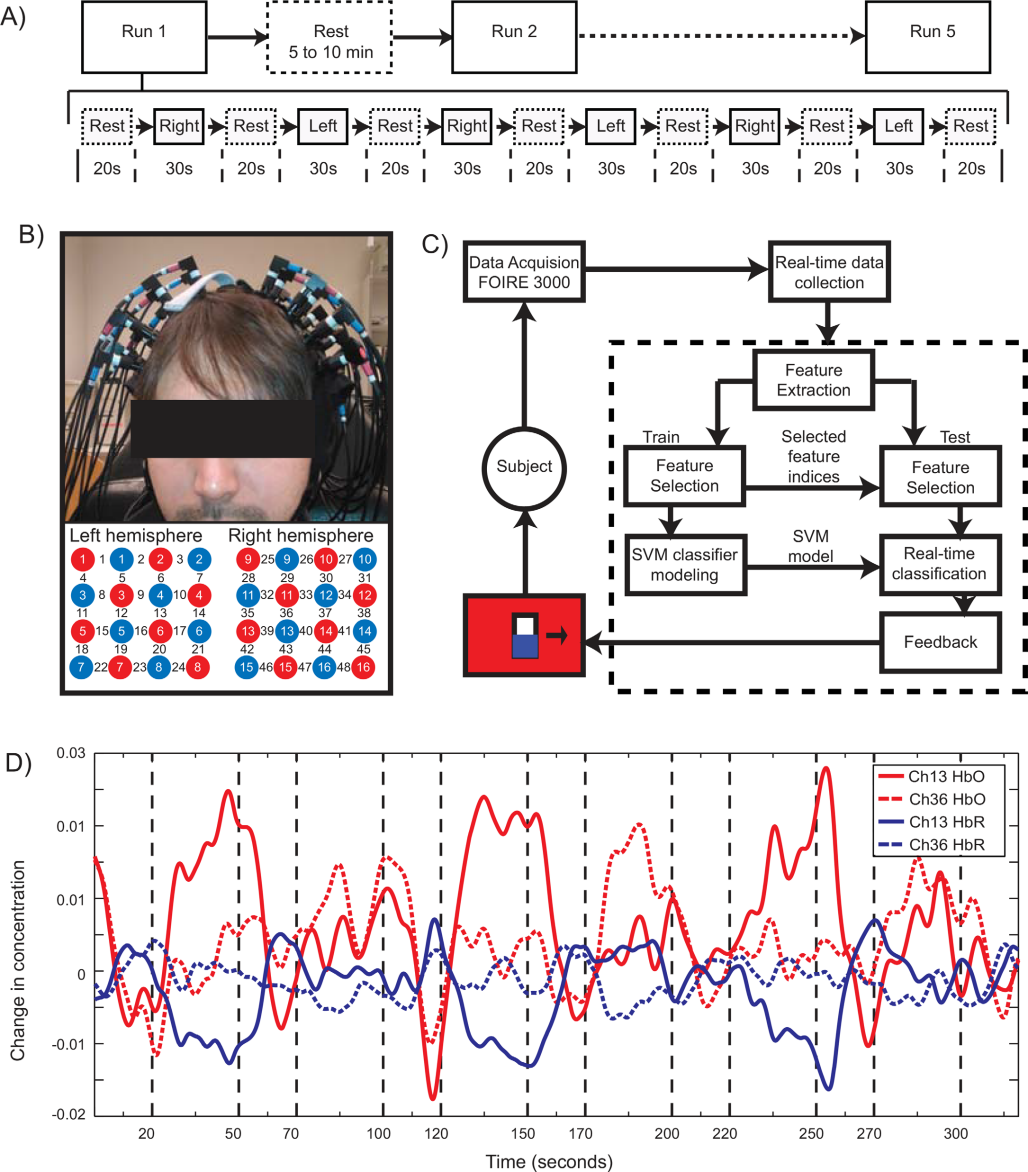
Overview of the real-time neuro-feedback binary classification system. A) Experiment protocol and timeline for the experiment: Runs 1–5 are separated by 5–10 minutes rest periods. The sequence of blocks with their durations for each Run is shown under Run 1. B) Arrangement of optodes and the headmount. The optodes are placed over the motor area and arranged in 4x4 checkerboard topography. The red and blue circles indicate emitters and detectors respectively. The numbers 1–48 indicate the recorded channels. C) The architecture of the designed system indicating its various functional units. The feedback generated by BCI is displayed to the subject as indicated. For details refer to text. D) Sample time course of activations during motor execution. The pre-processed data from Experiment 1, Subject S11, Run 3 is shown. Channel 13 and 36 are from PMC in left and right hemispheres. The contralateral activations of HbO and dip in HbR can be clearly identified from the plots.

To analyze brain activations during bilateral hand movement execution (ME) and imagery (MI), the experimental protocol was designed to consist of five conditions, namely movement execution of left and right hand, motor imagery of left and right hand, and rest condition. The participants were asked to perform repetitive hand movements similar to clenching and unclenching an imaginary ball at a frequency of ~1 Hz during motor execution. During movement imagery, participants were asked to imagine similar movements, without actually moving their hands. No physical movement was observed in any subject during the imagery tasks. Participants were asked to participate in five runs of one experiment, each of which consisted of six task blocks separated by seven rest blocks (Fig 1A). Some participants, however, did not complete all five runs. One participant, S11 performed both Experiments 1 and 2. A break of about 5–10 minutes was given between runs. In Fig 1A, Left and Right refers to left and right hand’s movement for both execution and imagery tasks. Each participant was seated in front of the screen that displayed the visual cues. As per the protocol, the cues for each block were as follows: a blue screen with a black dot for "Rest", a red screen with a Right arrow, for "Task-Right" and green screen with Left arrow, for "Task-Left". An activation-level meter (hereafter called thermometer as it is depicted graphically as a thermometer) with baseline level indicated at its middle, appeared on center of the screen during the training runs. For test runs, neurofeedback was given as the thermometer grades. The dynamic range of the thermometer was 20 units or levels.

Our study comprises three different experiment paradigms as shown in Table 1 and the details are given in this section. In all the experiments, the initial one or two runs were used for training the system. No feedback was provided during training runs, and the thermometer grade remained at the baseline. Following this, the subjects were instructed to perform the test runs with neurofeedback. Feedback was provided as increase or decrease in thermometer grade during correct and incorrect classification respectively.

**Table 1:** Overview of experimental design for various experiments.

	Run 1	Run 2	Run 3	Run 4	Run 5
**Experiment 1**	*ME*^*a*^	*ME*	ME	MI^b^	MI
**Experiment 2**	*MI*	MI	MI	MI	ME
**Experiment 3**	*ME*	MI	MI	MI	ME

^a^ME stands for Motor execution

^b^MI stands for Motor Imagery. Italicized runs represent the ones used to train the classifier.

#### Experiment 1

The main objective of this study was to implement real-time subject-dependent classification of bilateral hand movement using a movement execution trained BCI. The system was validated on bilateral motor execution and imagination data, to provide real-time classification results and spatial activation patterns for further analysis. In the experiment, the classifiers were adapted as per Eq (9) for the following test runs. Run 3 tested the classifier on ME for each subject. Runs 4 and 5 were used to test classification of MI based on ME models, and the subjects were asked to imagine the movements. In all the test runs, the subjects were provided a visual feedback based on the classification output.

#### Experiment 2

The objective of this study was to perform a corollary to Experiment 1, i.e., to implement real-time subject-dependent classification and feedback of left versus right hand motor imagery, based on a classifier built using covert hand movement data. In the MI runs, 1 to 4, the subjects (S21, S22, S23, S11) were instructed to imagine the movement they had practiced. The first run was used for training the classifier. For Runs 2 to 4, the classifier was updated after each Run, as per Eq (9). The performance of subjects performing MI was tested using the classifier and a neurofeedback was provided. Run 5 was used to test classification of ME based on MI models (with the classifier modeled based on the last two MI runs) and the subjects were asked to perform ME.

#### Experiment 3

The objective was to demonstrate the feasibility of a Subject-Independent Classifier (SIC) built from the ensemble data of all participants from Experiment 1, performing hand movement execution. At the beginning of this experiment, a practice session was provided where the subjects (S31, S32, S33, S34) were asked to perform hand clenching actions. During the experiment, in test runs 2, 3 and 4, the subjects were asked to perform MI of the practiced movements without moving their hands. In the ME run 5, the subjects were asked to execute the movement. Real-time classifications of overt and covert movements from new subjects were performed using the SIC and neurofeedback was provided in all the runs.

### Feature Extraction and Selection

The study aims to use multi-channel temporal information of changes in concentration levels of blood oxy hemoglobin (HbO) to classify volitional overt and covert hand movements. The smaller levels of concentration changes and lesser discrimination between movement classes offered by HbR, limits its use for further processing. The discriminative features from fNIRS recordings are extracted from the time averages of changes in HbO concentration from the various channels located over the motor cortex. The real-time classification of signal features and estimation of neurofeedback are performed at every unit time (1 second). Hence, for an *N*_*t*_ -channel arrangement, the features extracted at *k*^*th*^ second of a trial from n^th^ channel is given by,

$$f^{n}(k)=\sum_{i\in 1\;to\;f_{s}}\Delta HbO_{i}^{n}\;(k)$$ (1)

where *f*_*s*_ is the sampling frequency and *n = 1 to N*_*t*_. The feature set at *k*^*th*^ instant is given by,

$$F(k)=\left\{f^{1}(k),f^{2}(k),f^{3}(k),\ldots \ldots ,f^{N_{t}}(k)\right\}$$ (2)

A feature selection technique based on mutual information [6,7] selects *N < N*_*t*_ features from Eq (2). This technique effectively chooses the channels that provide optimal discriminating information for the task performed by the participant. For an *N*_*t*_*—*dimensional feature set *F*, the mutual information based technique selects, *S* ⊂ *F*, an *N*-dimensional subset that maximizes the mutual information, *I*(*F*;*ω*), where *ω* represents each class *i* ∈ {1,2}. Mutual information is given by,

$$I(F;\omega )=H(\omega )-H(\omega |F),\;\omega \in \left\{\omega_{1},\omega_{2}\right\}$$ (3)

$$H(\omega |F)=-\sum_{i=\left\{1,\;2\right\}}p(\omega_{i}|F)\;\log_{2}\;p(\omega_{i}|F)$$ (4)

where, *H*(*ω*) denotes the class entropy and *H*(*ω*|*F*) gives the conditional entropy. The conditional probability *p*(*ω*|*F*) is estimated using Parzen window method. The mutual information for all the *N*_*t*_ features are calculated and the best *N* features are selected to obtain,

$$S(k)=\left\{f^{n}(k)\right\},\;n\in selected\;N\;features$$ (5)

The value of *N* is set to 12 in this work, and it is ensured that equal number of features are selected from both left and right hemispheres. The performance of the system may vary depending on *N*, however, characterization of the effect of number of features on classification accuracy is beyond the scope of this work and hence would not be considered in this manuscript. The feature set *S(k)* for every *k*^*th*^ instant is fed as input to the classifier for real-time classification and to calculate neurofeedback.

### Support Vector Machines (SVM)

SVM is a supervised learning technique that creates a boundary between two classes of data based on a set of available training samples [8]. It designs a decision function that optimally separates the two classes in the training data. In this study we use a linear-SVM to separate left versus right hand movements. For real time classification, we consider the features obtained at each instant *k* as a separate training data sample. The data sample at *k*^*th*^ instant is the feature vector denoted by *S(k)* or *S*^*k*^. The SVM-classifier determines a weight vector *W*, that discriminates a class against the other by the projection *W'S* and linear discriminant rule,

$$\omega \;\{\begin{array}{c} \in \omega_{i}\;W'S^{k}\geq b\\ \in \omega_{i}\;W'S^{k}<b \end{array}$$ (6)

where *b* is a bias. This vector is determined by minimizing the cost function,

$$J(W)=\frac{1}{2}{\left\|W\right\|}^{2}$$ (7)

subject to the constraint,

$$Y^{k}\;(W'.S^{k}-b)\geq b,\;k=1\;to\;K$$ (8)

where *Y*^*k*^ is the class label corresponding to *S*^*k*^, that is a sample from the training data set {*S*^1^,*S*^2^,…..,*S*^*K*^} and *K* is the number of training data samples. The SVM classifier thus modeled is used to classify or to determine the label of incoming data samples.

### 1.3 Adapting Classifier and Feature selector

For neurofeedback training applications, the BCI is designed to provide feedback information regarding the quality of the performed task to the user in real time. Considering the non-stationarity of the neural signals there is a need to adaptively update the classifier and feature selector in the system [4,9].

In the subject-dependent classifier experiments, the initial run is used to select the most informative features and model the SVM classifier that optimally discriminates the binary class data. This is used to classify the data samples of Run 2 in real time. As given in (9), from the 3^rd^ Run onwards, the classifier is re-modeled using the data from two previous runs.

$${\left\{N;W\right\}}^{\left(r\right)}=\left\{\begin{array}{c} C(S^{\left(r-1\right)}),\;r=2\\ C([S^{\left(r-1\right)};S^{\left(r-2\right)}]),\;r>2 \end{array}\right\}$$ (9)

where, *r* is the run number, *S*^*(r)*^ is the data set collected during Run *r* and *C* denotes the feature selection and classifier modeling functions. Moreover, a bias cancellation is performed from Run 2 onwards that subtracts the average of SVM output during the Rest block from the following Task block. The real-time system thus adopts a between-runs adaptive strategy of re-training classifiers after each run and within-run adaptive bias correction of SVM outputs.

### Participants

The data were recorded from 11 healthy participants (both male and female, aged 21–35). All participants signed a written informed consent. The study was approved by the Institutional Review Board, Faculty of Medicine of the University of Tuebingen, Germany. Each participant was compensated monetarily for participation in the experiment.

### Data Acquisition

FNIRS signals were acquired using a Shimadzu FOIRE-3000 imaging system operating at a sampling rate of 7.69Hz, using wavelengths of 780nm and 830nm from laser sources. Emitters and detectors were separated by 25mm, and were placed on top of the participant’s head using a semi-flexible head mount. Sixteen sources and detectors were arranged in two 4-by-4 checkerboard topographies, as shown in Fig 1B centered on C3 and C4 of the International 10–20 System. This arrangement covered most of the primary motor, pre-motor and somatosensory cortices.

### Real-Time fNIRS-BCI System Schematic

The architecture of the real-time system designed is shown in Fig 1C. FNIRS signals are received online in the BCI-processing computer from the FOIRE-3000 equipment. The BCI-processing system consists of a feature extractor, a feature selector and a classifier. The data are fed into the processing system in real-time. As indicated in Section 1.1, we extract the relevant features from the recorded fNIRS data. The data from training runs are used to select the informative features and to model the classifier as explained in Section 1.3. For the test runs, after the movement task stimulus onset, a bias correction is performed and the extracted features are classified in real-time using the SVM model created. The classified output is generated at every second so as to provide feedback in real time. This output is presented to the participant in the form of a graphical thermometer in which a correct classification would lead to a unit rise in the thermometer, and incorrect classifications would lead to a unit fall in the thermometer reading. The thermometer reading remains at 0 (middle) during “Rest” period and returns to this position at the end of every movement task.

### Offline Data Analysis

The preprocessing steps used to improve the Signal-to-Noise ratio and derive optimal information from recorded fNIRS data were as follows: the data was baseline corrected followed by pre-coloring using a hemodynamic response function-low pass filter; the global trends were removed using Wavelet-Minimum Description Length technique. A sample time course of activation of pre-processed fNIRS recording is shown in Fig 1D, which plots the HbO and HbR signals from channels 13 and 36 (corresponding to primary motor cortex (BA4) in left and right hemispheres respectively) from Subject S11, Experiment 1, Run 2 From the figure, distinct changes can be seen in the contralateral activity of oxy- and deoxy-hemoglobin concentrations. These changes were utilized for feature extraction and modeling of the SVM-based classifier.

To ensure stationarity of the training data used to create classifiers, 5- fold cross-validation analysis was performed. The training data was randomly split into five subsets. In each cross-validation fold, data from four subsets were used to select features and model classifier that was used to classify the remaining test subset. The process was repeated to test all the subsets and an average performance over all the folds was calculated. The low values of training classification accuracy’s standard deviations indicated the low variance of the training dataset used (not shown).

fNIRS signals were also analyzed to determine statistically significant spatial activations by a univariate approach using SPM 5 fNIRS toolbox [10]. The spatial plots of mutual information obtained from Eq (4) and the SVM outputs obtained from Eq (8) are also reported among the various results.

## Results

The evaluation results of the real time fNIRS based neurofeedback system for classifying left versus right hand overt and covert movements are presented in this section. Subsection 3.1 indicates the real time classification performance of the system and the system parameters identified by offline analyses on the data, followed by 3.2 explaining the subject independent classifier, its parameters and the results obtained. Subsection 3.3 reports the results indicating homologous activations during overt and covert movements using spatial activation maps. The practical significance of each of the results is also discussed.

### Real-Time Classification

**Fig 2:**
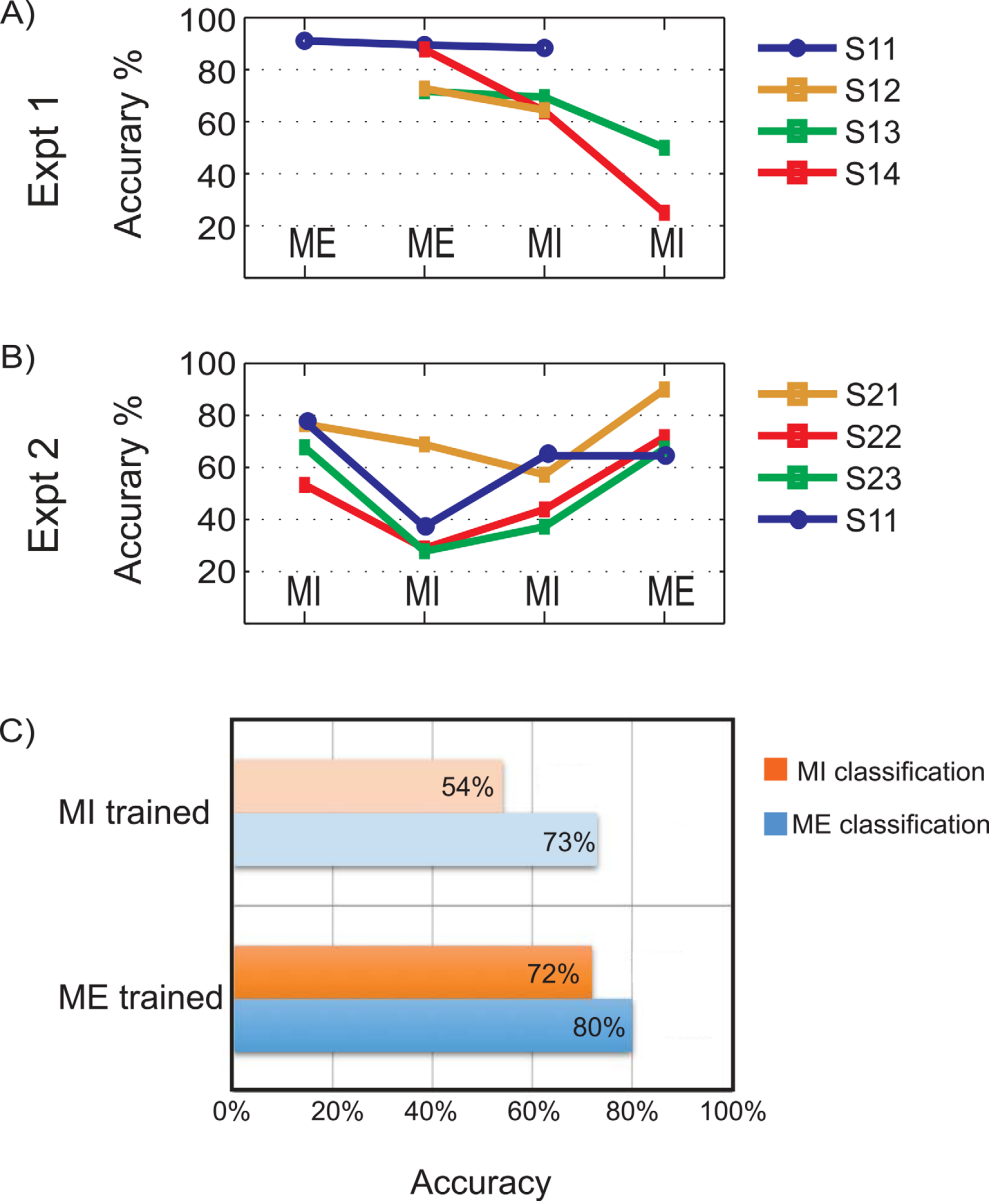
Real-time classification performance for experiments 1 and 2. A and B) The percentage classification accuracies for online binary (right v/s left motor tasks) classification for 7 subjects for the Experiment 1 (A) and Experiment 2(B) are shown. The motor tasks involved are right and left Motor Execution (ME) and Motor Imagery (MI). Note: Subject S11 was common between both experiments 1 and 2. C) Comparison of mean classification accuracies of motor imagery and execution when classifier was trained on either motor imagery or motor execution.

The motor performance of subjects is evaluated in real-time by online feature extraction and SVM classification of bilateral motor tasks and the percentage classification accuracies are reported. Fig 2 summarizes the performance of the proposed real-time classification system for overt movement execution and imagery with neurofeedback. The results indicated are percentage classification accuracies attained by subjects in each of the runs for various tasks indicated using MI (motor imagery) and ME (motor execution) labels. To comply with experimental guidelines, subjects were allowed to discontinue the experiment if they experienced fatigue.

The *Experiments 1* and *2* used subject-dependent classifier models for bilateral MI and ME classification. In *Experiment 1*, the average classification accuracy over four subjects obtained for runs 3 and 4 are 80% (ME) and 72% (MI) respectively, where the task performed is indicated within brackets. Not all subjects were able to complete the five runs due to fatigue. In *Experiment 2*, for all subjects the accuracy falls after the first run and improves afterwards. On an average, the classification accuracies are reported as 69% (MI), 41% (MI), 51% (MI) and 73% (ME) for runs 2, 3, 4 and 5 respectively. The last run (run 5) used the classifier trained on MI for online classification of bilateral ME. A general trend seen in the results is a dip in performance after the first run, followed by gradual rise. Although the paradigm we use is insufficient to prove the effect of neurofeedback training and its learning effect in subjects, the performance trend obtained indicates subject's capability to identify and enhance motor control strategy after each run. Longer experiment sessions might reveal more information on such a learning curve. The simple adaptive strategies of re-training classifier and bias correction seem to work efficiently in this real-time system.

#### Classification parameters

**Fig 3:**
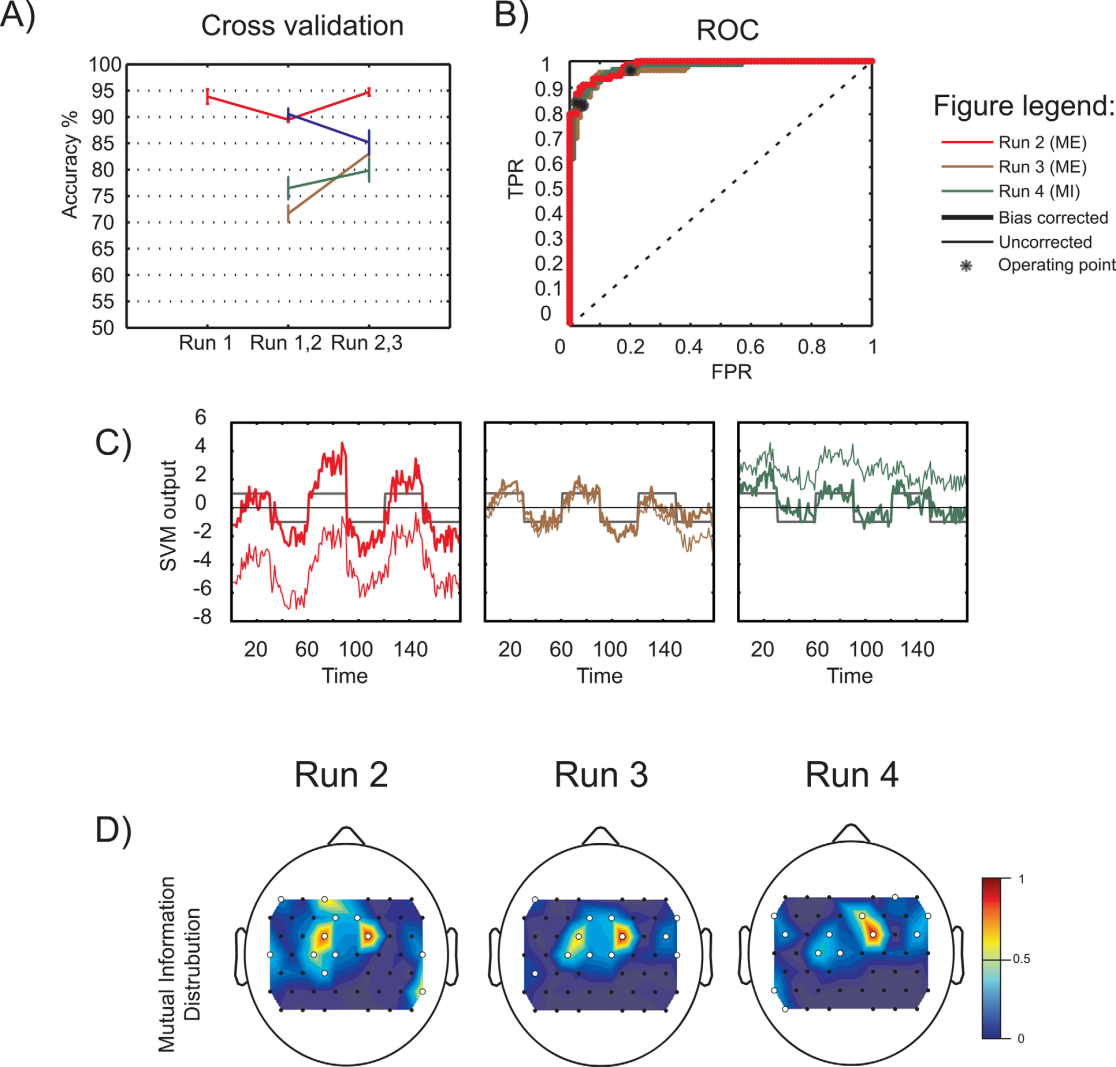
Classifier parameters for Experiment 1. A) The offline 5-by-5 cross-validation classification accuracies in percentage. The x-axis indicates the runs that contribute to the dataset, which is later used to generate the online classifier model. The error bars represent standard deviations. B) ROC curves of the classifiers used in the different runs during online classification. The operating point is indicated by an asterisk (*). C) The SVM outputs obtained from the online classification. The uncorrected and bias-corrected values are represented using thin and thick lines respectively. The class of data is indicated as +1 for right hand and -1 for left hand blocks. D) The spatial distribution of mutual information for each of the training dataset. The white dots represent selected channels based on high mutual information.

**Fig 4:**
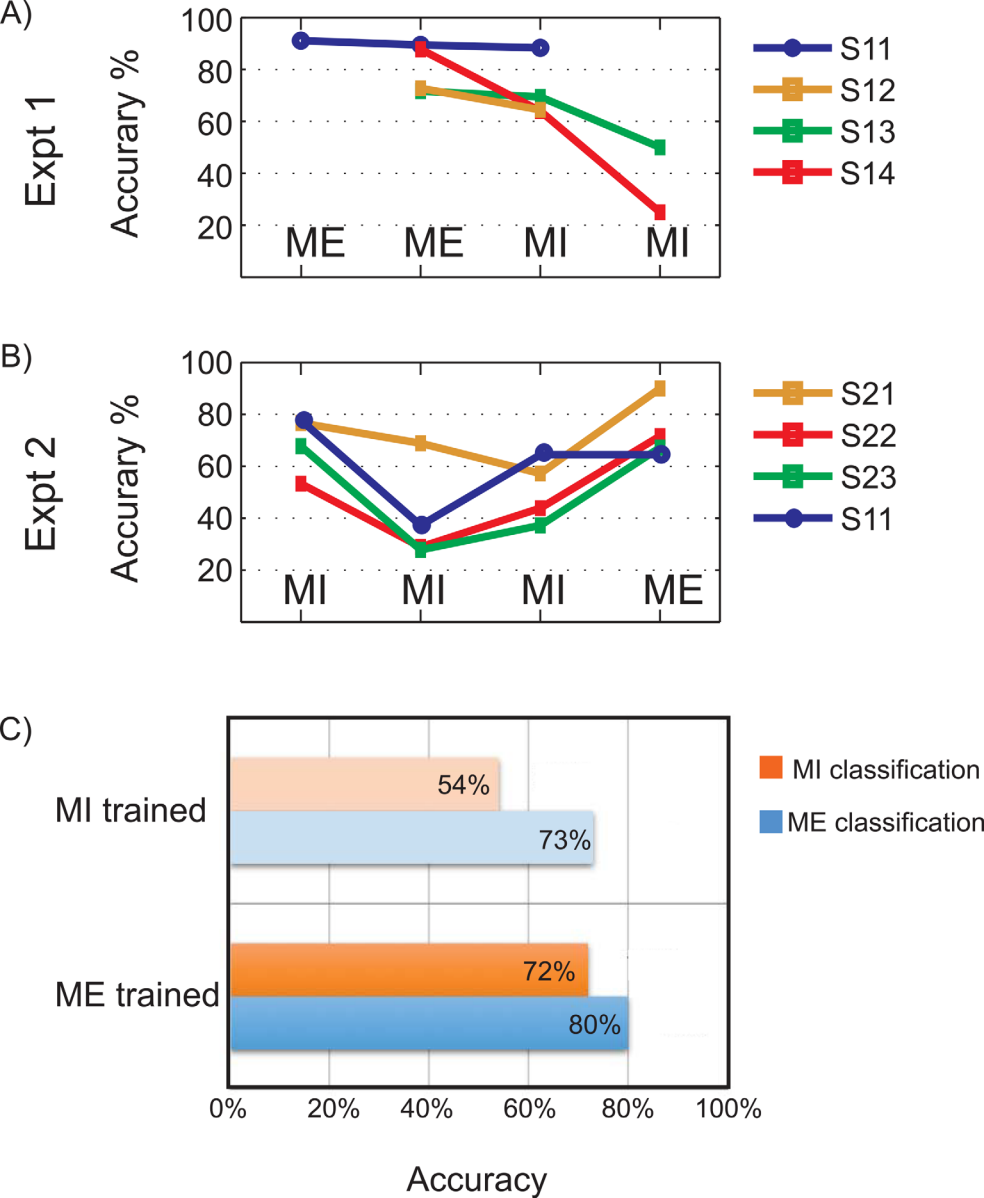
Classifier parameters for Experiment 2. A) The offline 5-by-5 cross-validation classification accuracies in percentage. The x-axis indicates the runs that contribute to the dataset, which is later used to generate the online classifier model. The error bars represent standard deviations. B) ROC curves of the classifiers used in the different runs during online classification. The operating point is indicated by an asterisk (*). C) The SVM outputs obtained from the online classification. The uncorrected and bias-corrected values are represented using thin and thick lines respectively. The class of data is indicated as +1 for right hand and -1 for left hand blocks. D) The spatial distribution of mutual information for each of the training dataset. The white dots represent selected channels based on high mutual information.

The intermediate results in the online system, namely, the data used for modeling the classifiers, the classifier models created, and the feature selection proceedings were evaluated. The results are shown in Figs 3 and 4. The objectives were: (1) to demonstrate offline classification accuracies, (2) to obtain various parameters of the SVM classifier used and identify how they contribute to the classification performance, (3) to illustrate how mutual information based feature selection helps extract the optimal information and (4) to show the temporal averages over runs demonstrating class-dependent hemodynamic activity.

**Fig 5:**
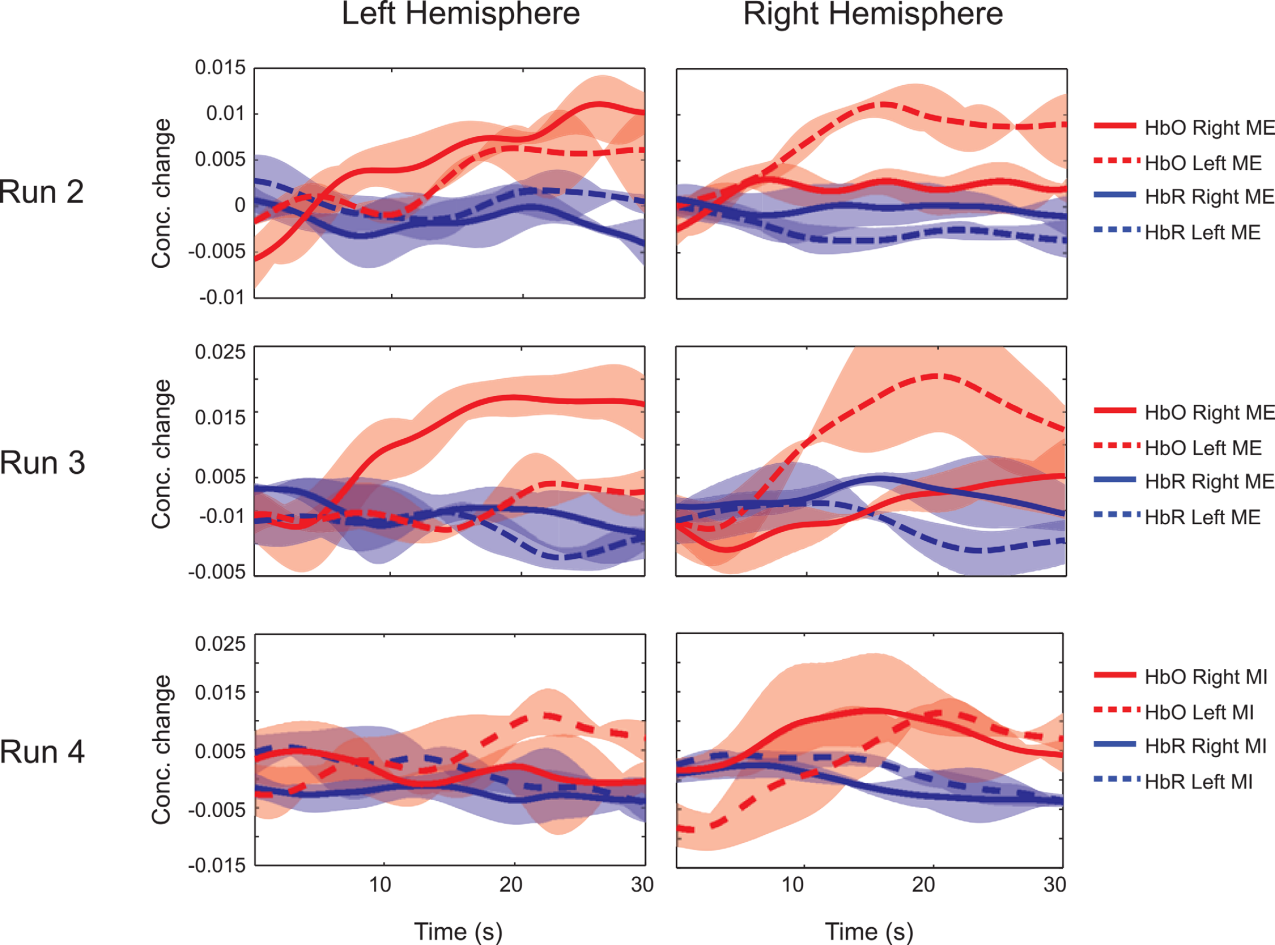
Average time series of activations in Subject S11 during Experiment 1. Runs 2, 3, 4 indicate activation levels given by concentration changes of HbO and HbR averaged over trials and selected channels. The channel selection is based on mutual information. The discriminative contralateral activations during both classes of movement can be noted.

**Fig 6:**
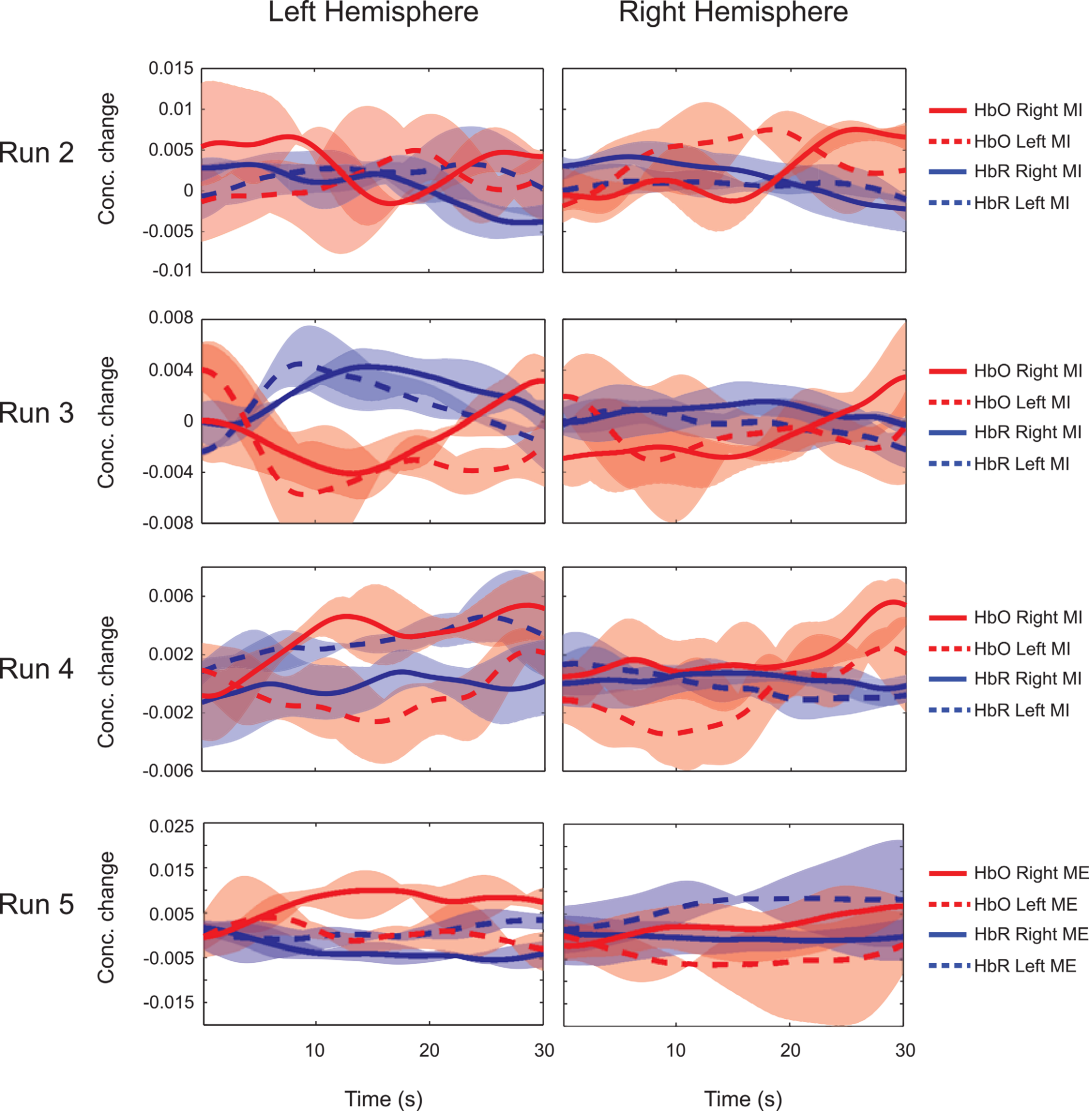
Average time series of activations in Subject S11 during Experiment 2. Runs 2–5 indicate activation levels given by concentration changes of HbO and HbR averaged over trials and selected channels. The channel selection is based on highest mutual information between left and right blocks. Note: Y-axis labels are different for different runs.

The results for analyses (1)-(4) in experiments 1 and 2 are illustrated in the following subsections. Fig 3 and Fig 5 represent Experiment 1, and Fig 4 and Fig 6 indicate results of Experiment 2 respectively.

Fig 3A indicates the cross-validation accuracies for offline classification of ME data in *Experiment 1* used to create SVM models. The average accuracies are 82.06±1.31% and 85.70±1.59% respectively (classifier modeling using Run 1 was not performed for all the subjects). As seen, the standard deviation for each subject indicated in the curves is less than 2%. This indicates a uniform performance in all folds of cross validation and so, the entire data are used to create classifiers. To demonstrate the various characteristics of the system, data from Subject S11 is used as an example. Fig 3C indicates the SVM output obtained in the real-time classification of data for all the runs. The curves clearly indicate the advantage of using bias correction for real-time classification. In all the runs, the SVM output gradually increases and reaches its peak value after 3 to 5 seconds from the onset of the movement cue. This can be identified as an effect of hemodynamic delay. However, in this experiment this delay is not taken into account by the classifier as it provides real-time output right from the start of movement onset. Fig 3D displays the spatial distribution of mutual information obtained based on oxy-hemoglobin data. The channels with higher mutual information are indicated by white dots and the features from these channels are selected for further classification. The channels providing highest mutual information of bilateral movement are obtained from the primary motor cortex of the brain. Fig 3B illustrates the receiver operating characteristics (ROC) curves of the various classifiers used. The classifier operation points (indicated by the black asterisk) are at high True Positive Rate (TPR)—low False Positive Rate (FPR) regions, which indicate good classification performance.

The cross-validation accuracies obtained from classifier-training data for various runs of *Experiment 2* are shown in Fig 4A. The offline classification results reported are for MI classification. The mean over subjects for classifier models 1 to 4 are obtained as 84.11 ± 2.69%, 73.75 ± 2.47%, 74.17 ± 2.11% and 77.29 ± 2.21% respectively. The data recorded from subject S11 is used for further analyses and the results are reported. The SVM output obtained for every sample-classification in real-time for all the four runs are reported in Fig 4C. Fig 4D displays the mutual information distribution in the recorded channels. Comparing the activation maps, it can be noted that in all runs, except run 3, activations are observed over the pre-motor and motor areas. However, it can be noted that the patterns are as not localized as during ME. The ROC curves, obtained from each classifier, are shown in Fig 4B. The classifier used in run 2 generates a good ROC with operating point in high TPR–low FPR region providing an accuracy of 78% in real-time classification. But the ROC curve for Run 3 indicates a bad classifier design. This is reflected in the low classification accuracy (37%) obtained using this classifier (Fig 4B, brown curve). The classifier ROC improves later for runs 4 and 5 with better trend and operating points yielding better accuracy (64.44% in both runs).

Fig 5 (*Experiment 1*) and 6 (*Experiment 2*) explain the temporal changes in hemodynamic activity (HbO and HbR) averaged over selected channels in both hemispheres. The data are averaged over all the trials in each run and the upper and lower limits of the data are displayed along with their mean. The discriminating temporal activity in terms of contralateral activations can be seen in the curves. For example, in Fig 5, Run 3, for right hand ME, HbO levels show activations only in the left hemisphere. Similarly, in Run 3, HbO levels in right hemisphere rises as a result of activation during left hand movement. In Fig 6, the plots of Runs 2 to 4 and Run 5 correspond to MI and ME task respectively. It can be noted that, the levels of HbO concentration changes and the discrimination capabilities between MI classes are lesser when compared to ME data.

### Subject-Independent Classification

**Fig 7:**
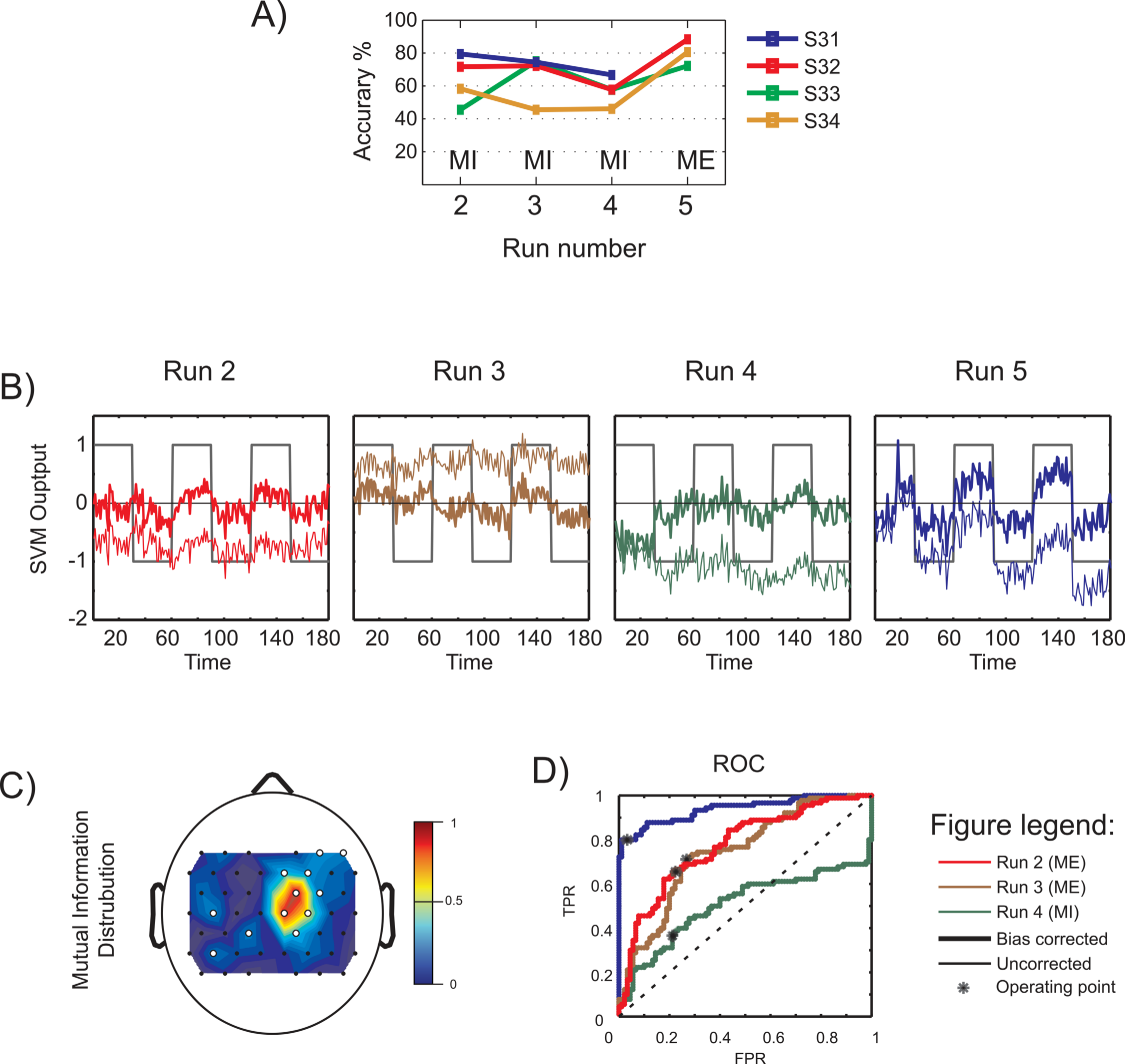
Subject-independent classification results and parameters. A) Online classification accuracies for each run. B) The SVM outputs obtained from the online classification. The uncorrected and bias-corrected values are represented using thin and thick lines resp. The class of data is indicated as +1 for right hand -1 for left hand blocks. C) The spatial distribution of mutual information for the ensemble data training set. The white dots indicate channels with high mutual information and hence selected. D) ROC curves of the classifiers used in the different runs of online classification. The operating point is indicated by an asterisk (*).

The major focus of *Experiment 3* was to construct a subject-independent classifier (SIC) trained on multi-subject ME data, which could be used as a generalized classifier for MI and ME in real-time from new subjects. The ME data from Runs 1 to 3 of Experiment 1 was used to train an SVM subject-independent classifier. 5-fold cross-validation is performed to identify non-stationarity in data and a test accuracy of 68.22±3.25% was obtained. Due to the low variation in each of the cross-validation folds, the entire data were used to construct a subject-independent classifier model with a training accuracy of 68%. The features selected by the system are mentioned in *Section 3*.*2*.*3*. The real-time classification accuracy values obtained are displayed in Fig 7A. Out of four subjects, the performance of S34 was poor for MI classification which brought down the average accuracy. The subject reported difficulty imagining movements as they were tired and distracted. The mean classification accuracies for runs 2 to 5 were 64% (MI), 67% (MI), 57% (MI) and 80% (ME) respectively. The results prove the applicability of the proposed subject-independent pattern classifier for real-time neurofeedback movement classifications.

#### SIC System parameters

**Fig 8:**
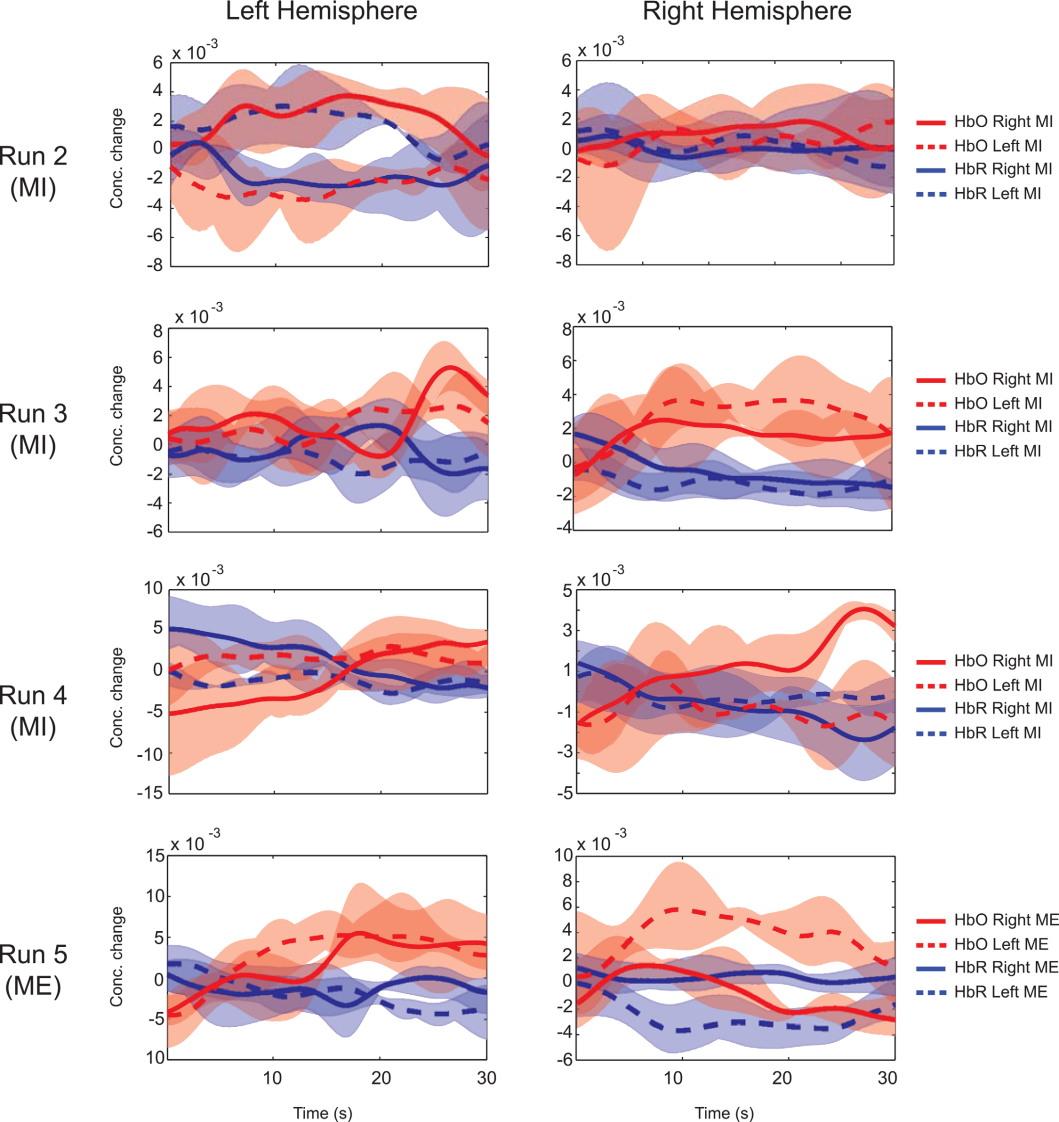
Average time series activations in Subject S32 during Experiment 3. Runs 2–5 indicate activation levels given by concentration changes of HbO and HbR averaged over trials and selected channels. The channel selection is based on highest mutual information between left and right blocks. Note: Y-axis labels are different for left and right panels for better visualization of differences.

The detailed analysis of the subject-independent classifier generated in our study to classify MI and ME data is reported in this section and the data from Subject, S32 is used. Fig 7B shows the SVM outputs generated in four experimental runs. The advantage of using bias correction is evident from the plots. The spatial distribution of mutual information for the data used to train the classifier is shown in Fig 7C. Even though slightly shifted towards right hemisphere, the channels in the motor area are found to report high mutual information (indicated by white dots) and are hence selected for classification. The ROC curves for the classifiers used in all the four runs are given in Fig 7D. Runs 2, 3 and 5 indicate good ROC and selection of operating points, yielding real time classification accuracies of 72%, 72% and 88% respectively. Run 4 fails to perform well due to the bad ROC and hence could give only 58% classification accuracy. The temporal HbO activity during MI and ME for the data collected in *Experiment 3* are reported in Fig 8. The results are similar to previous experiments. The level of HbO concentration change is less for MI compared to ME, and the activation curves for both right hand and left hand MI overlap in most cases, providing minimum discrimination.

## Discussion

The real-time neurofeedback system using our signal processing strategy offers the following advantages: (1) the system identifies the optimal discriminative features based on mutual information and applies these for classifier modeling, (2) the classifier adapts by itself after each run, making use of the data collected in the previous run, and (3) the bias correction within runs compensates for the dc shift in the feature space to provide better classification performance. The intermediate results of feature selection and classification and were reported in the previous section. The channels chosen using mutual information based feature selection are found to lie over the motor cortex in most of the cases. The bias correction that provides an intra-run classifier adaptation clearly results in better classification accuracies. The performance of the binary classifiers used in various runs is demonstrated using ROC curves, with the operating point defining the threshold at which the system uses the classifier model. The runs with good classification accuracies generate almost ideal ROC curves, with their operating point in the high TPR-low FPR region. Each of the parameters are inter-related, and together, they define the real-time system.

**Fig 9:**
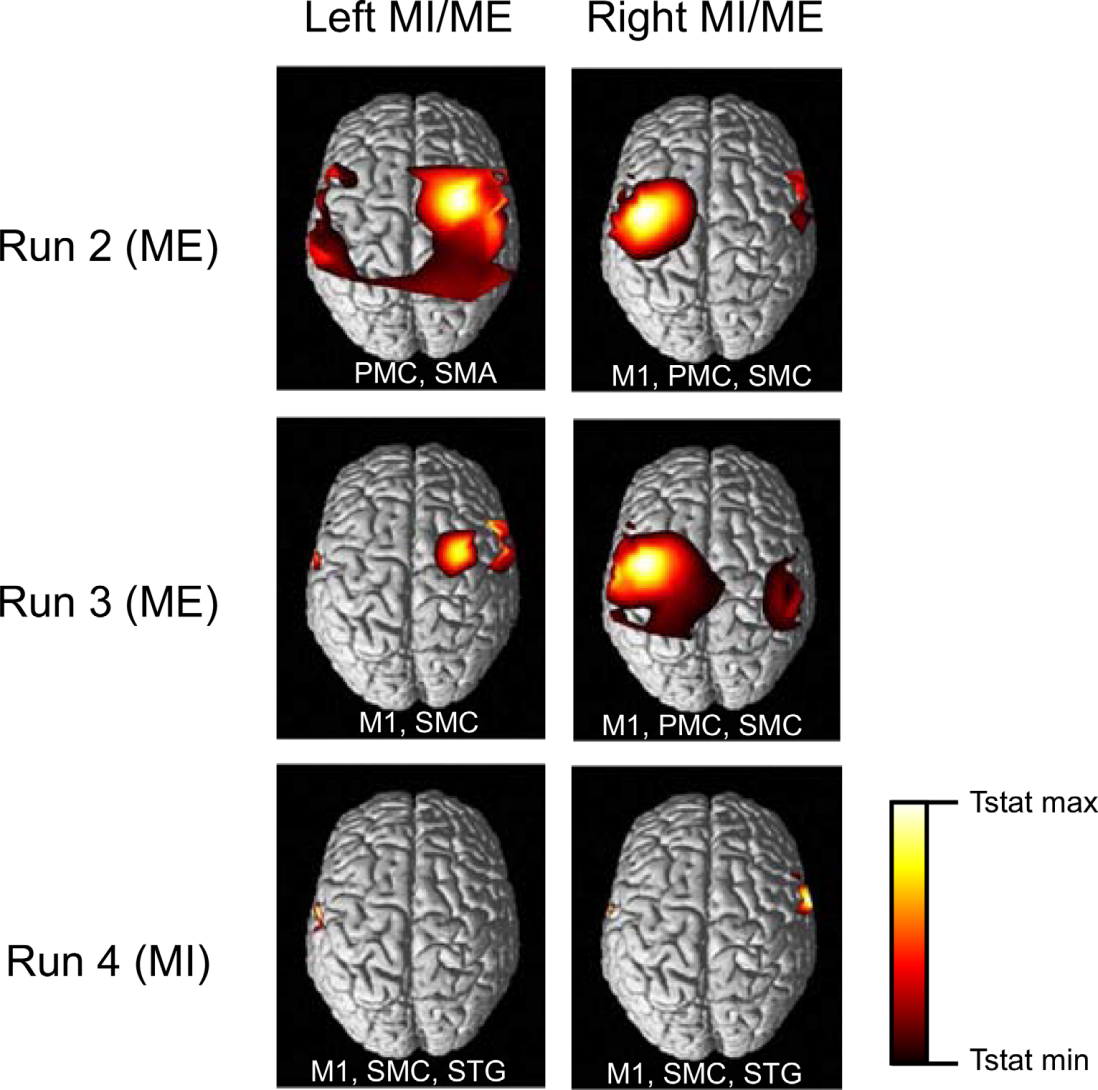
Experiment 1: Thresholded t-statistics maps. The statistically significant activations (p<0.001) during bilateral ME and MI for different runs are as shown. Abbreviations of the major areas activated in the contralateral hemisphere are written within each panel. The major areas activated include PMC: Pre-Motor Cortex, M1: Primary Motor Cortex, SMA: Supplementary Motor Area. MI activated superior temporal gyrus (in addition to others) in the final run.

**Fig 10:**
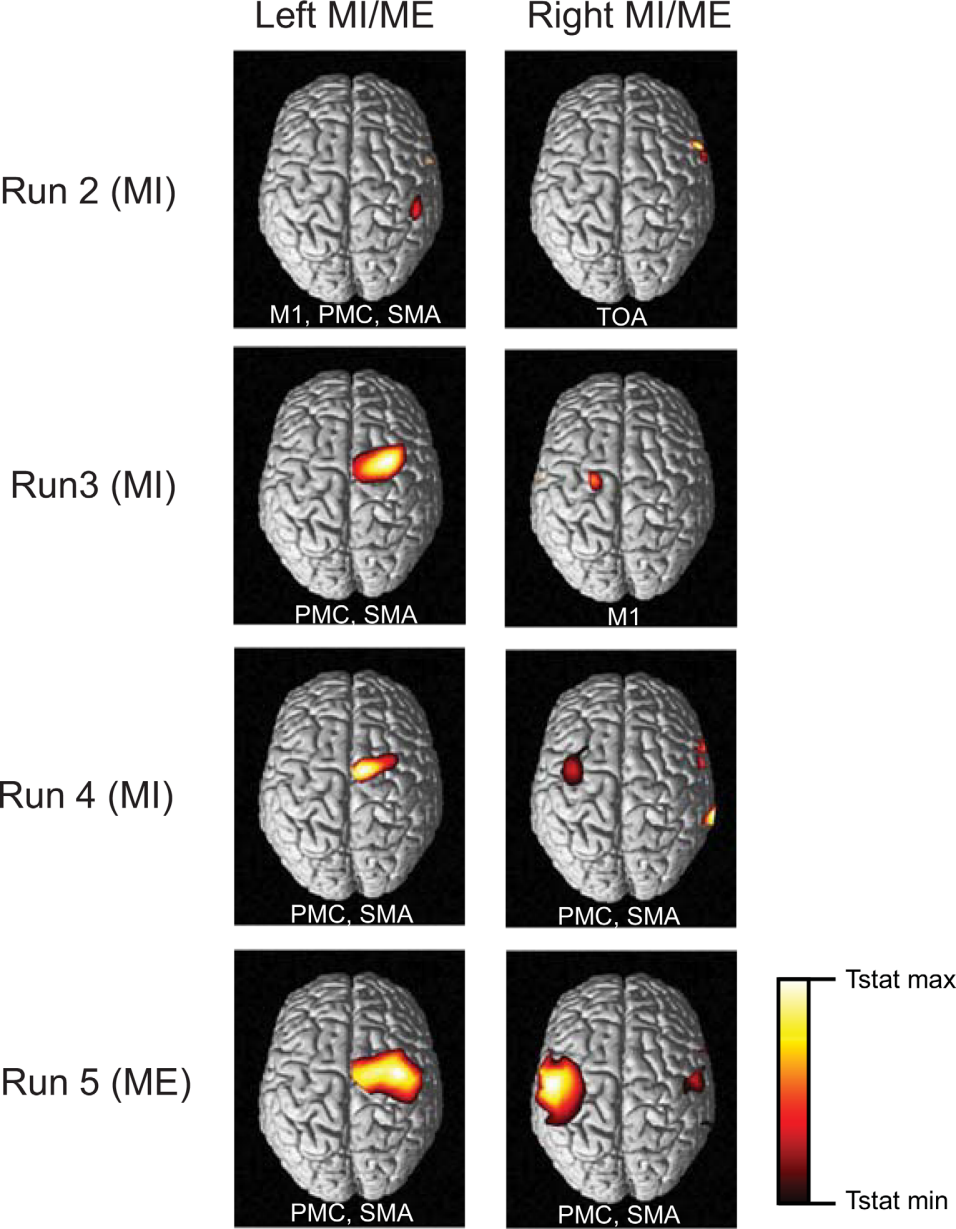
Experiment 2: Thresholded t-statistics maps. The statistically significant activations during bilateral MI and ME for different runs are as shown. Abbreviations of the major areas activated in the contralateral hemisphere are written within each panel. MI activates somatosensory cortex and temperopolar areas (TOA) in addition to Primary Motor Cortex (M1), Supplementary Motor Area (SMA) and Pre-Motor Cortex (PMC). For the MI runs the activations are for p<0.05 and for ME the activations are for p<0.001.

**Fig 11:**
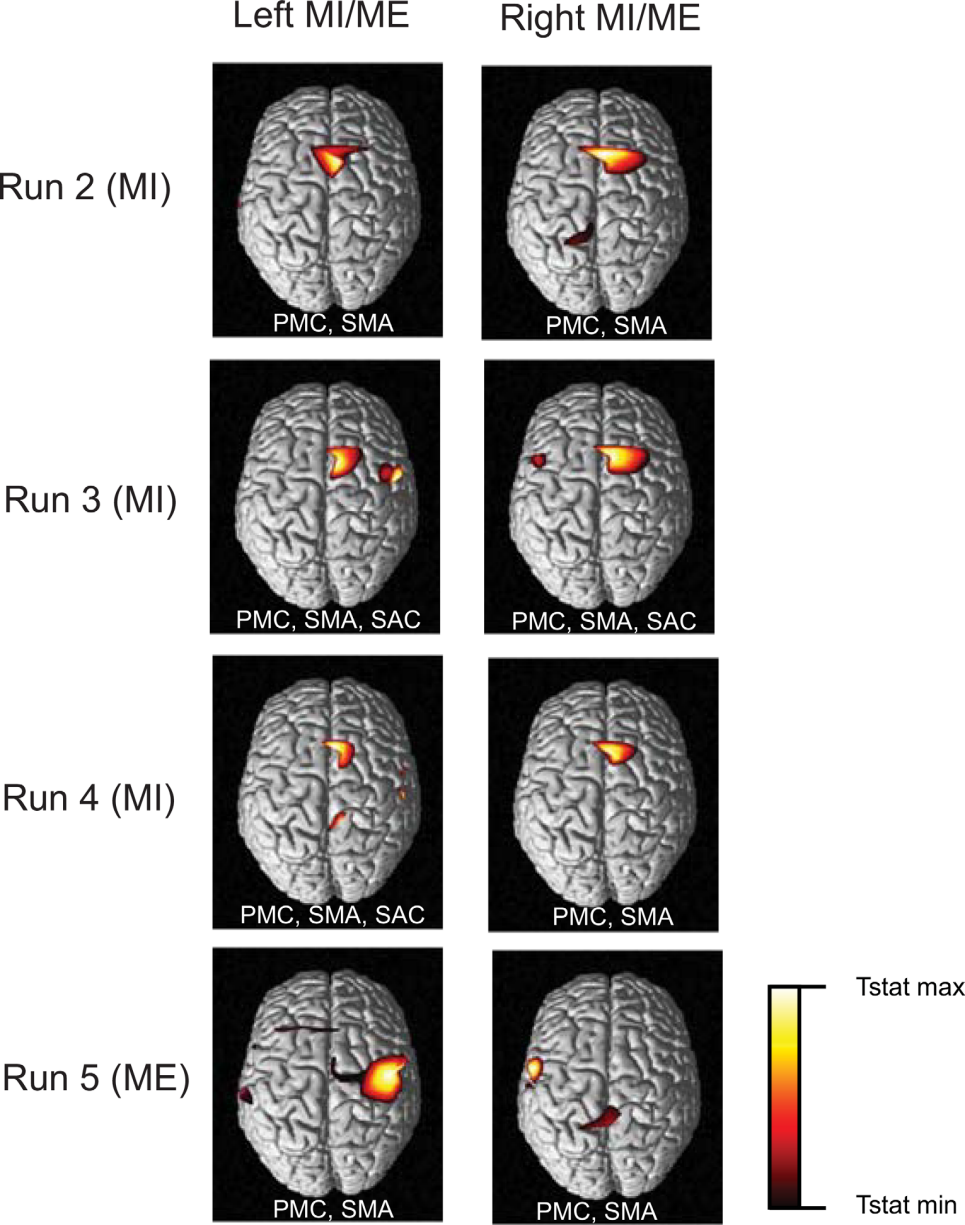
Experiment 3: Thresholded t-statistics maps. The statistically significant (p<0.001) activations during bilateral ME and MI for different runs are as shown. Abbreviations of the major areas activated in the contralateral hemisphere are written within each panel. In addition to Pre-Motor Cortex (PMC), Supplementary Motor Area (SMA) and Primary Motor Cortex (M1), primary somatosensory cortex (S1) was also activated during motor imagery runs as shown.

The change in levels of HbO and HbR during ME and MI have been reported in earlier studies [4,11]. In our study, the time averages over the selected channels in both hemispheres are shown in Figs 5, 6 and 8. ME/MI is accompanied with activations in the contralateral hemisphere that reflect as a rise in HbO and a dip in HbR levels. For ME, we demonstrate a distinct representation of hemodynamic activity in various hemispheres for different runs. For MI, the discrimination is not as good as ME, due to lower levels of activation. The time series of the hemodynamic activity in these selected features is shown. Figs 9, 10 and 11 show the activation maps as heat-maps along with the channels used for feature selection. Interestingly, when contrasted with ME, the MI runs had activations that were more anterior and apical, localizing around the SMA. An earlier study comparing fMRI-BOLD activations between MI and ME in a similar task reports similar activations, with ME leading to larger activations in the M1 (primary motor cortex) and MI leading to higher activations in the SMA that localize towards the apical portion of the brain [12]. Although it appears that our results provide supporting evidence to this report, our interpretation cannot be conclusive due to the technical difficulties of proper co-registration of fNIRS optodes with the corresponding structural MRI of each subject.

Furthermore, the study is expected to bring us a step closer to assist stroke-patient rehabilitation using the subject-independent classifier with real-time neurofeedback. The study in healthy subjects can potentially be applied to patients with more optimizations. Also, the subject-independent motor activation patterns from healthy subjects can be used to train patients with motor disabilities to imitate and later even generate similar patterns. Studies testing the feasibility of using BCIs for stroke rehabilitation found statistically and clinically significant gains in motor control and functional tasks in response to motor learning combined with functional electric stimulation (FES); [13,14]. Though these studies were quite promising, normal motor control and function were not completely attained, and not all subjects had significant improvement. Current theories in motor learning and instrumental conditioning of the brain responses propose a novel approach of closed-loop BCI for effective recovery of movement [15], by conditioning spatio-temporal patterns of the brain responses associated with the desired movement, concurrently with the afferent sensory input induced by peripheral stimulation of the muscles of the limbs that produces movement. A hypothesis that can be tested with real-time subject-independent classification and feedback is that: by training patients to produce normal brain activity, one may be able to influence brain plasticity that results in normal brain function and motor behavior. In the case of motor function, this strategy is supported by evidence that practicing movements that are as close to normal as possible might help to improve motor function [16], by guiding newly sprouting axons to the appropriate cortical regions [17]. The development of a subject-independent classifier based BCI is thus an important step towards successful stroke rehabilitation.

The results obtained in our experiments suggest the applicability of our technique for neurofeedback experiments. However, the system is still open to modifications in areas such as robustness, usability etc. The subject-independent classifier model, even though its performance is as good as subject-dependent classifiers, can still be optimized by incorporating head size, shape and activation pattern information from the individual subjects involved. The usability indicates the preparation time for the data acquisition and the extent to which subject can wear the equipment. Currently, it takes a long time for setting up the head cap, as it involves managing the obstructing hair from the optical path. Most of the subjects reported discomfort after wearing the cap for over 45 minutes, and pointed this out as a reason for failing to do well in the motor task. These are limitations in the current study and we intend to look into further possibilities to address them in the future. Furthermore, future studies should look at comparing the brain and behavioral changes induced by neurofeedback training with subject-independent classification and subject-dependent classification in a controlled manner so that the advantages and disadvantages of the methods are identified. Here, we present a preliminary step to extract optimal features in a motor activity and apply it on a simple adaptive classification system to provide better binary classifications. Concluding the subject's capability to regulate his own brain activity using neurofeedback is thus beyond the scope of this study.

## Conclusions

In conclusion, this study primarily focuses on real-time binary classification of left versus right hand movement execution and imagery using a SVM based classifier. A subject-independent pattern classifier generated from movement execution data using the feature extraction and selection strategy discussed above was used in real-time classification of MI and ME. The neuronal activity correlates between MI and ME were explored and utilized to create a generic classifier. The performance of the system in terms of bilateral movement classification accuracies obtained in various sessions of the different subjects are reported. The classifier parameters obtained in each of the experiments conducted, indicating robust and accurate performance, are separately discussed. The data are analyzed offline to identify the spatial and temporal activations and the results were also demonstrated. The results are promising, and we hope to overcome and address the various drawbacks of the current system, and to develop a real-time BCI system for clinical rehabilitation purposes.
