# Correction: Real-Time Subject-Independent Pattern Classification of Overt and Covert Movements from fNIRS Signals

**DOI:** 10.1371/journal.pone.0162567

**Published:** 2016-09-01

**Authors:** Neethu Robinson, Ali Danish Zaidi, Mohit Rana, Vinod A. Prasad, Cuntai Guan, Niels Birbaumer, Ranganatha Sitaram

There is an error in affiliation 6 for author Ranganatha Sitaram. Affiliation 6 should be: Institute for Biological and Medical Engineering, and Department of Psychiatry and Division of Neuroscience, Schools of Engineering, Biology & Medicine, Pontificia Universidad Católica de Chile.

Ranganatha Sitaram is not affiliated with #1, #2, or #3 but only with #6: Institute for Biological and Medical Engineering, and Department of Psychiatry and Division of Neuroscience, Schools of Engineering, Biology & Medicine, Pontificia Universidad Católica de Chile.
